# Tooth loss and rehabilitation in the elderly: an intersectional analysis of gender, race, and territory

**DOI:** 10.1590/1980-549720260020.supl.1

**Published:** 2026-07-24

**Authors:** Ana Karine Macedo Teixeira, Raul Anderson Domingues Alves da Silva, Alice Castro Guedes Mendonça, Kenio Costa de Lima

**Affiliations:** I Universidade Federal do Ceará - Fortaleza (CE), Brazil.; II Universidade Christus - Fortaleza (CE), Brazil.; III Universidade Federal do Rio Grande do Norte - Natal (RN), Brazil.

**Keywords:** Intersectional framework, Tooth loss, Oral health, Health inequalities

## Abstract

**Objective:**

To assess tooth loss, dental function, and rehabilitation in older adults in 2010 and 2023, and its intersection with gender, race, and territory.

**Methods:**

A sample of elderly individuals from the 2023 National Oral Health Survey (SB Brasil) (n=9,745) and 2010 (n=7,619) was used. The outcomes were: tooth loss, dental functionality, and oral rehabilitation in the elderly population. Demographic data (sex, race, age, and Brazilian regions) were used as independent variables. Poisson regression was performed for each oral health variable based on an interaction model between race/color and gender, stratified by region.

**Results:**

The oral health status of the elderly population improved between 2010 and 2023, with reduction in edentulism (from 53.4% to 36%), and increase in functional dentition (from 13.2% to 28.6%) and complete anterior dentition (from 12.4 to 21.2%). The use of prostheses in elderly individuals with fewer than 20 teeth did not improve during the evaluated period. In the intersectional analysis, black women presented a higher prevalence of edentulism (PR=1.16; CI 1.04-1.29), lower functional dentition (PR=0.74; CI 0.66-0.83), and lower anterior dentition (PR=0.85; CI 0.78-0.93) compared to white men, while black men were the least rehabilitated (PR=0.83; CI 0.78-0.90). Gender and racial inequalities varied across regions. The North region, despite having the worst oral health conditions among the elderly population, did not show inequality in functional dentition and edentulism

**Conclusion:**

Despite advances in oral health among elderly Brazilians, inequalities persist, marked by the intersection of gender, race, and territory, with a more severe impact on black women.

## INTRODUCTION

The persistence of oral diseases highlights the existence of profound inequities and economic inequalities worldwide^1^ ^,^ ^2^. In Brazil, the demographic transition has resulted in rapid population aging^1^ ^,^ ^3^, making the health of older adults, including oral health, a relevant aspect for public policies^1^.

Tooth loss, especially edentulism, is a direct consequence of prolonged exposure to oral diseases, reflecting the accumulation of health experiences throughout life, and is considered a marker of oral health in older individuals^1^ ^,^ ^3^. Aging is a significant risk factor, with the peak of severe tooth loss occurring in elderly people^1^ ^,^ ^4^. Although Brazil has recorded a reduction in the prevalence of edentulism among individuals aged 65-74 years^1^, approximately 35% of this population is still edentulous, exceeding the estimated global average^1^. Edentulism is associated with severe consequences, including impaired chewing, nutrition, quality of life, and a higher risk of all-cause mortality, even after adjustment for socioeconomic and health factors^1^ ^,^ ^3^ ^,^ ^4^.

To understand the unequal distribution of tooth loss, it is essential to consider the social determinants^1^, and the lens of intersectionality provides the necessary theoretical framework to explore the complexity of health among vulnerable populations^2^ ^,^ ^5^. The concept of intersectionality, rooted in the black feminist movement^5^, proposes that axes of social oppression-such as gender, race/skin color, and social class-interact and overlap, resulting in different levels of vulnerability and exclusion in health^2^ ^,^ ^5^. Applying this perspective is crucial to ensuring the right to dignified aging and for public policies to recognize the inequities that prevent many groups from reaching the age of 60^5^.

Evidence in Brazil shows that reductions in tooth loss did not occur uniformly, being more pronounced among white individuals and those with higher levels of education^1^. Structural racism and sexism are recognized as social mechanisms that drive inequalities^1^ ^,^ ^6^. From the perspective of intersectionality, this evidence cannot be understood in isolation, but rather through the overlap of axes of social oppression throughout these individuals’ life trajectories, which led some groups to have greater access to oral health care than others.

In particular, the intersectionality between race and gender reveals heightened vulnerability^6^. Although gender may be a highly important factor, the impact of social mechanisms such as racism and sexism may be more pronounced in women’s oral health^2^.

Another aspect that interacts with the outcome of tooth loss is territory. Evidence shows that living in rural areas has a negative impact on the oral health quality of older Brazilian adults^7^ ^,^ ^8^, but it is still unknown how the intersection of race and gender affects the oral health of older adults across different regions of the country.

In this context, it is important to recognize that, despite advances in the country’s Oral Health Policy, inequalities persist^9^. The oral health condition of the black population, including quilombola communities^10^, may partly be explained by limited access to health services due to the lack of adequate public transportation, insecurity, or insufficient financial resources^11^. When it comes to older adults, these issues become even more relevant, as this population traditionally faces difficulties related to transportation and mobility^12^.

Therefore, monitoring tooth loss and dental functionality, as well as rehabilitation among older Brazilian adults, while broadening the understanding of overlapping inequalities through the lens of intersectionality across Brazilian regions, is essential for identifying the most vulnerable groups and contributing to the development of health interventions and policies that are more equitable and capable of overcoming structural social barriers that perpetuate oral health inequities^1^ ^,^ ^2^. Thus, the aim of the study was to evaluate tooth loss, dental functionality, and prosthetic rehabilitation among older adults in 2010 and 2023 and their gender-race-territory intersection.

## METHODS

This cross-sectional, population-based study was conducted using secondary data from the National Oral Health Survey (SB Brasil) 2023 and 2010, made available by the Ministry of Health. In 2010, the sample consisted of 32 domains, including state capitals and inland municipalities across the country, with participants selected through cluster sampling. In 2023, the survey covered 53 domains, including states and capitals. The sampling design allowed estimates to be produced for the five Brazilian regions in both editions. Detailed methodological aspects of SB Brasil are available in other publications^13^ ^,^ ^14^ ^,^ ^15^.

Data collection involved interviews and oral examinations conducted at participants’ homes by oral health professionals from the Brazilian Unified Health System (SUS), who were trained and calibrated to ensure the consistency of the examinations. The National Research Ethics Committee approved both surveys (2010: protocol no. 15,498; 2023: protocol no. 4,823,054), and participants signed an informed consent form.

For this study, the sample consisted of older adults aged 65 to 74 who participated in both editions, totaling 7,619 in 2010 and 9,745 older adults in 2023.

Six outcomes were used to measure oral condition according to tooth loss and functionality, as well as prosthetic rehabilitation in the elderly population, considering the examination criteria of the World Health Organization (WHO) and the technical manuals of SB Brasil^13^ ^,^ ^14^ ^,^ ^16^: 1) DMFT Index (mean number of permanent teeth with caries experience - decayed, missing, and filled teeth); 2) Mean number of missing teeth (mean number of permanent teeth lost that compose the DMFT); 3) Edentulism (yes/no) - loss of all teeth, including teeth lost for reasons other than dental caries -; 4) Functional dentition (yes/no) - presence of 20 or more teeth in the mouth, without considering the presence of occluding pairs -; 5) Complete anterior dentition (yes/no) - presence of all teeth in the second and fifth sextants -; 6) Use of prosthesis (yes/no) - use of some type of dental prosthesis by elderly individuals who have fewer than 20 teeth.

As independent variables, the following demographic variables were used: sex (male/female), race/color (White/Black), age (65-69 years / 70-74 years), Brazilian regions (North, Northeast, Southeast, Central-West, South), and type of city (capital city/interior).

The intersectional gender-race analysis was conducted only on the 2023 sample. For the definition of gender, the variable “female” was used for women and “male” for men. In the racial analysis, the black population was considered based on self-identification as mixed-race (“parda”) and black (“preta”). Individuals identified as Indigenous, Asian, or with missing race/color information were excluded from the analysis (final 2023 sample = 9,414). The racial categorization is consistent with the framework of collective health epidemiology and with the guidelines of Brazilian public policies^17^.

The analysis was conducted using Stata 13.0 software, employing the survey module to account for the sampling design and weights derived from the sampling process. Initially, a descriptive analysis of the demographic and oral health variables (tooth loss and dental functionality, and prosthetic rehabilitation) was performed for the years 2010 and 2023, presenting prevalence estimates and their respective 95% confidence intervals (95% CI). Subsequently, the hypothesis of association between oral health variables and demographic variables was tested using Pearson’s chi-square test, with a significance level of 5%. For the intersectional analysis, Poisson regression was used for each oral health outcome (edentulism, functional dentition, anterior dentition, and prosthesis use), including an interaction model between race/color and gender variables. Finally, the intersectional analysis for each dependent variable was stratified by region. Prevalence Ratios (PR) with 95% Confidence Intervals (95% CI) were used for all analyses.

## Data Availability Statement:

All the data supporting the findings of this study originate from the National Oral Health Survey (SB Brasil 2023) and are available upon request from the Brazilian Ministry of Health. The dataset is not publicly available because it contains sensitive information that may compromise the privacy and confidentiality of the survey participants, and access is therefore subject to authorization by the responsible authority.

## RESULTS

The characterization of the sample of older adults participating in SB Brasil in the years 2010 and 2023 maintained the same pattern, with the majority composed of females, white individuals, aged 65 to 69 years, residing in the Southeast region and in inland areas of the country. However, an increase was observed in the number of black older adults residing in the Northeast region and in inland areas between the two surveys (Table 1).

**Table 1.** Distribution of demographic variables in elderly people. SB Brasil 2010 and 2023.

	2010 % (CI)	2023 % (CI)
Sex		
Female	62.2 (58.7-65.6)	60.5 (58.2-62.7)
Male	37.8 (34.4-41.3)	39.5 (37.3-41.8)
Race		
White	55.8 (51.5-60.0)	50.3 (46.5-54.1)
Black	44.2 (40.0-48.5)	49.7 (45.9-53.5)
Age (years)		
65 to 69	54.9 (51.5-58.2)	56.1 (52.4-59.8)
70 to 74	45.1 (41.8-48.5)	43.9 (40.2-47.6)
Region		
North	5.1 (3.5-7.5)	5.6 (4.8-6.5)
Northeast	9.8 (8.0-11.8)	24.1 (21.4-27.0)
Southeast	62.8 (57.3-68.1)	46.9 (42.6-51.2)
Central-West	6.1 (4.8-7.6)	6.7 (5.3-8.6)
South	16.2 (13.2-19.7)	16.7 (14.3-19.3)
City type		
Capital	32.1 (25.3-39.8)	23.8 (21.4-26.3)
Interior	67.7 (60.2-74.7)	76.2 (73.7-78.6)

Regarding oral health status (Table 2), the DMFT index decreased from 27.5 (SD = 0.25) in 2010 to 23.5 (SD = 0.31) in 2023, with a reduction in the average of nearly five missing teeth. Edentulism, in turn, decreased from 53.4% to 36.5%, and there was an improvement in dental functionality, considering functional dentition and complete anterior dentition, which reached prevalences of 28.6% and 21.2% in 2023, respectively. However, the prevalence of denture use among older adults with fewer than 20 teeth remained at similar levels between 2010 (84.2%) and 2023 (85.3%).

**Table 2.** Prevalence of oral health variables (tooth loss, functionality, and rehabilitation) in elderly individuals. SB Brasil 2010 and 2023.

	2010	2023
	Mean (SD)	Mean (SD)
DMFT	27.5 (SD=0.25)	23.5 (SD=0.31)
Mean number of missing teeth	25.2 (SD=0.38)	19.8 (SD=0.49)
	% (CI)	% (CI)
Edentulism	53.4 (49.4-58.0)	36.5 (33.4-39.6)
Functional dentition	13.2 (11.0-15.8)	28.6 (25.5-32.0)
Complete anterior dentition	12.4 (10.0-15.3)	21.2 (18.9-23.8)
Use of prostheses in elderly people with fewer than 20 teeth.	84.2 (81.3-86.7)	85.3 (82.5-87.6)

Women showed higher edentulism (38.5%), lower dental functionality (25%), and lower complete anterior dentition (19.4%) than men, but also a higher prevalence of prosthesis use (87.8%). The 70-74 age group had greater tooth loss, but showed no difference in prosthetic rehabilitation compared to the 64-69 age group. Black older adults had more missing teeth than white older adults; however, black individuals (81.6%) were less rehabilitated than white individuals (89.3%). The Southeast region had more older adults with 20 or more teeth (35.3%) and complete anterior dentition (25.7%), while the South region showed the highest prevalence of older adults using some type of prosthesis (92.2%). Furthermore, older adults living in rural areas (37.8%) had higher edentulism than those living in capital cities (32.4%) (Table 3).

**Table 3.** Prevalence of oral health variables (tooth loss, functionality, and rehabilitation) according to demographic characteristics. SB Brasil 2023.

	Edentulism % (CI)	Functional dentition % (CI)	Complete anterior dentition % (CI)	Use of prosthesis % (CI)
Sex				
Male	33.4 (30.0-37.1)	34.1 (30.1-38.4)	24.0 (20.7-27.7)	80.8 (76.7-84.3)
Female	38.5 (34.4-42.7)*	25.0 (21.6-28.8)**	19.4 (16.6-22.5)*	87.8 (85.1-90.1)**
Age (years)				
65 to 69	31.5 (27.8-35.4)	32.5 (28.7-36-6)	23.7 (20.8-26.8)	86.1 (82.7-89.0)
70 to 74	42.8 (38.8-47.0)**	23.7 (19.7-28.6)**	18.0 (14.8-21.8)*	84.3 (80.8-87.2)
Race/color				
White	34.5 (30.0-39.6)	33.5 (28.6-38.7)	25.0 (21.4-29.1)	89.3 (66.9-74.8)
Black	38.6 (35.5-41.7)	23.9 (21.0-27.1)**	17.6 (15.4-20.1)**	81.6 (77.5-85.1)**
Region				
North	39.9 (35.3-44.8)	18.1 (13.7-236)	14.0 (9.8-19.6)	82.4 (77.4-86.5)
Northeast	37.6 (34.0-41.4)	21.9 (18.3-26.1)	18.7 (158-22.0)	78.0 (72.4-82.7)
Southeast	35.2 (29.6-41.4)	35.3 (29.5-41.6)**	25.7 (21.4-30.6)**	86.8 (81.3-90.8)
Central-West	40.5 (35.4-45.7)	23.8 (19.9-28.1)	17.4 (14.5-20.6)	88.6 (83.8-92.1)
South	35.5 (30.2-41.3)	25.0 (20.6-29.9)	16.1 (13.4-19.2)	92.2 (88.9-94.6)**
City type				
Capital	32.4 (29.4-35.4)	30.5 (28.0-33.1)	23.6 (21.5-25.8)	85.6 (83.3-87.7)
Interior	37.8 (33.9-41.8)*	28.0 (24.0-32.4)	20.5 (17.5-23.8)	85.1 (81.6-88.1)

*p<0,05; **p<0,001.

In the intersectional gender-race analysis (Table 4), it was observed that older black women presented the worst oral health condition for all tooth loss indicators. The prevalence of edentulism among black women was 1.16 (CI 1.04-1.29) times higher than among white men, who presented the best condition of functional dentition compared with all other gender-race categories, with greater inequality observed for black women (PR=0.74, CI 0.66-0.83). Regarding complete anterior dentition, white men differed only from black women, who showed a lower proportion of teeth in the anterior region (PR=0.85, CI 0.78-0.93). In prosthesis use, white women presented the best condition compared with all other categories, including black women (PR=0.91, CI 0.86-0.96), while black men were the least rehabilitated (PR=0.83, CI 0.78-0.90).

**Table 4.** Intersectional analysis of oral health variables (tooth loss, functionality, and rehabilitation) according to gender and race/color. SB Brasil 2023.

Gender-Race/color	n	Edentulism		Functional dentition		Complete anterior dentition		Use of prosthesis	
		% (CI)	PR (CI)	% (CI)	PR (CI)	% (CI)	PR (CI)	% (CI)	PR (CI)
White Man	1,322	31.1 (25.7-37.1)	1	41.0 (34.2-48.1)	1	28.4 (22.8-34.8)	1	84.3 (78.0-89.0)	0.91* (0.85-0.98)
White woman	2,277	36.8 (31.1-42.9)	1.09 (0.97-1.21)	28.5 (23.6-33.9)	0.83** (0.74-0.92)	22.8 (18.5-27.7)	0.93 (0.84-1.02)	92.0 (88.4-94.5)	1
Black man	2,276	35.5 (31.6-39.6)	1.07 (0.96-1.18)	28.1 (23.4-33.3)	0.82* (0.70-0.95)	20.2 (16.3-24.8)	0.89* (0.81-1.00)	77.2 (71.7-81.9)	0.83** (0.78-0.90)
Black woman	3,539	40.6 (36.3-45.0)	1.16* (1.04-1.29)	21.2 (17.5-25.5)	0.74** (0.66-0.83)	16.0 (13.1-19.3)	0.85** (0.78-0.93)	84.2 (79.9-87.7)	0.91* (0.86-0.96)

*p<0,05; **p<0,001.

When the region was added to the intersectional gender-race analysis (Table 5), different patterns were observed for each region. Although the North region has the worst indicators of tooth loss in the country, it did not show gender-race inequalities for edentulism, functional dentition, or complete anterior dentition. Regarding rehabilitation, it was found that white women use dental prostheses more frequently, while black older adults in the North region were less likely to receive rehabilitation (PR=0.89, CI 0.83-0.94).

**Table 5.** Intersectional analysis of oral health variables (tooth loss, functionality, and rehabilitation) according to gender, race and region. SB Brasil 2023.

Region	Gender-Race/color (N)	Edentulism		Functional dentition		Complete anterior dentition		Use of prosthesis	
		% (CI)	PR (CI)	% (CI)	PR (CI)	% (CI)	PR (CI)	% (CI)	PR (CI)
North (n=2,165)	White man (165)	42.1 (29.5-55.9)	1	17.2 (8.4-31.9)	1	10.5 (5.6-18.8)	1	83.9 (74.6-90.2)	0.89* (0.81-0.98)
	White woman (258)	41.7 (28.0-56.8)	0.99 (0.67-1.46)	17.6 (11.4-26.0)	1.01 (0.87-1.15)	14.0 (8.1-23.1)	1.04 (0.96-1.15)	94.2(86.4-97.7)	1
	Black man (759)	38.5 (32.1-45.3)	0.94 (0.72-1.22)	18.6 (14.3-23.8)	1.01 (0.88-1.17)	14.6 (10.3-20.3)	1.04 (0.95-1.15)	76.8 (70.5-82.2)	0.81** (0.76-0.87)
	Black woman (983)	40.2 (33.7-47.0)	0.96 (0.76-1.22)	18.0 (10.8-28.5)	1.01 (0.85-1.19)	14.3 (7.4-25.7)	1.04 (0.92-1.18)	84.0 (78.2-88.5)	0.89* (0.83-0.94)
Northeast (n=3,135)	White man (263)	28.3 (19.4-39.4)	1	32.7 (21.1-46.8)	1	27.4 (16.9-41.1)	1	82.7 (72.4-89.8)	0.98 (0.86-1.14)
	White woman (573)	45.2 (37.2-53.4)	1.31* (1.06-1.61)	20.3 (13.6-29.0)	0.89 (0.68-1.05)	22.2 (15.5-30.8)	0.93 (0.76-1.15)	83.9 (76.7-89.2)	1
	Black man (850)	35.4 (29.4-41.8)	1.10 (0.95-1.29)	28.3 (20.6-37.4)	0.93 (0.75-1.16)	21.4 (14.7-30.0)	0.93 (0.75-1.13)	70.9 (61.4-78.9)	0.84* (0.74-0.97)
	Black woman (983)	39.2 (34.1-44.6)	1.18* (1.01-1.38)	15.9 (12.7-19.7)	0.80* (0.66-0.96)	13.6 (10.8-17.1)	0.84* (0.71-0.99)	79.2 (71.9-85.0)	0.94 (0.85-1.05)
Southeast (n=1,418)	White man (280)	31.3 (23.1-41.0)	1	47.8 (37.5-58.4)	1	34.4 (26.0-43.9)	1	81.5 (69.8-89.4)	0.89 (0.79-1.03)
	White woman (432)	34.7 (25.0-45.9)	1.05 (0.88-1.27)	34.2 (25.8-43.9)	0.79* (0.65-0.96)	26.6 (19.0-35.7)	0.89 (0.74-1.07)	90.8 (83.3-95.1)	1
	Black man (239)	31.8 (24.9-39.7)	1.01 (0.85-1.19)	34.6 (25.8-44.7)	0.79 (0.60-1.05)	24.4 (17.1-33.5)	0.87 (0.71-1.06)	85.2 (73.4-92.3)	0.94 (0.83-1.06)
	Black woman (467)	40.8 (32.8-49.4)	1.16 (0.96-1.39)	25.9 (18.5-35.0)	0.70** (0.57-0.85)	18.7 (13.2-25.8)	0.81** (0.70-0.93)	86.3 (77.4-92.0)	0.95 (0.86-1.05)
Midwest Central-West (n=1,299)	White man (210)	22.3 (14.1-33.4)	1	42.7 (30.9-55.3)	1	26.9 (17.7-38.7)	1	87.7 (79.6-92.8)	0.94 (0.86-1.04)
	White woman (337)	45.7 (33.2-58.8)	1.43** (1.06-1.93)	21.5 (15.4-29.2)	0.73* (0.56-0.94)	14.2 (9.4-20.8)	0.85* (0.73-1.00)	93.2 (84.9-97.1)	1
	Black man (304)	44.5 (32.5-57.2)	1.40** (1.08-1.82)	16.5 (10.5-24.8)	0.68** (0.53-0.88)	15.7 (10.8-22.3)	0.87* (0.75-1.00)	84.0 (72.9-91.1)	0.90* (0.81-0.99)
	Black woman (448)	39.6 (27.4-53.2)	1.26** (1.01-1.63)	23.6 (16.0-33.3)	0.75* (0.60-0.93)	18.0 (12.7-24.8)	0.89 (0.74-1.07)	88.2 (82.2-92.4)	0.95 (0.88-1.01)
South (n=1,397)	White man (404)	32.4 (25.3-40.5)	1	29.5 (23.3-36.5)	1	16.2 (11.2-22.7)	1	89.3 (81.7-94.0)	0.91** (0.85-0.97)
	White woman (677)	34.3 (28.3-40.8)	1.03 (0.91-1.15)	23.9 (18.4-30.4)	0.93 (0.84-1.02)	18.3 (14.4-23.0)	1.03 (0.95-1.11)	97.7 (94.8-99.0)	1
	Black man (127)	37.9 (24.5-53.4)	1.08 (0.85-1.38)	24.9 (15.1-38.0)	0.93 (0.80-1.09)	10.0 (5.5-17.7)	0.93 (0.85-1.03)	71.1 (52.1-84.8)	0.73** (0.5-0.92)
	Black woman (192)	46.6 (34.8-58.8)	1.26 (0.97-1.63)	17.8 (12.3-25.0)	0.85* (0.76-0.96)	9.6 (5.7-15.7)	0.93 (0.85-1.01)	92.4 (82.5-96.9)	0.95 (0.88-1.02)

*p<0,05; **p<0,001.

Elderly women in the Northeast, both white (PR = 1.31, CI 1.06-1.61) and black (PR = 1.18, CI 1.01-1.38), are more edentulous than white men. Regarding functional dentition (PR = 0.80, CI 0.66-0.96) and complete anterior dentition (PR = 0.84, CI 0.71-0.99), black women showed worse conditions when compared to white men from the Northeast. It was also observed that black men use fewer prostheses (PR = 0.84, CI 0.74-0.97).

In the Southeast, gender-race inequality was observed in functional dentition (PR = 0.70, CI 0.57-0.85) and complete anterior dentition (PR = 0.81, CI 0.70-0.93), with black women presenting the worst condition. No gender-race inequalities were observed regarding prosthesis use in this region.

In the Central-West region, white men maintain lower tooth loss when compared with all gender-race categories. White women showed a higher prevalence of edentulism (PR = 1.43, CI 1.06-1.93), and black men had the lowest functional dentition (PR = 0.68, CI 0.53-0.88). Regarding the use of dental prostheses, black men are the least rehabilitated (PR = 0.90, CI 0.81-0.99).

Finally, the South region maintains the pattern of inequality for black women only in functional dentition (PR = 0.85, CI 0.76-0.96); and, similarly to other regions, elderly black individuals use dental prostheses less frequently (PR = 0.73, CI 0.57-0.92). Still regarding prosthetic rehabilitation, white men in the South also use prostheses less frequently (PR = 0.91, CI 0.85-0.97) than white women.

## DISCUSSION

The present study demonstrated that, although advances occurred between 2010 and 2023 for the elderly population, these improvements were not distributed equitably. Racism and gender issues intersect with population aging, with significant repercussions for oral health across all regions of the country. Elderly black women remain the most vulnerable group regarding tooth loss and dental functionality, while elderly black men show a lower prevalence of prosthesis use.

While the marked reduction in DMFT and edentulism may indicate progress in oral health policy and access to dental services^1^, the increase in functional dentition points to a potential improvement in masticatory efficiency. In turn, the increase in complete anterior dentition reflects improvements in complementary oral functions, such as incisal function, phonetics, and aesthetics-factors directly related to self-perception of oral function and the quality of life of older adults.

However, the use of prostheses remained practically stable throughout the evaluated period, suggesting limitations in the provision of prostheses or the persistence of barriers to access specialized care for older adults. This scenario may be related to the accumulated demand for treatment resulting from the high prevalence of tooth loss among older adults^18^, associated with the low availability of other types of prostheses besides complete dentures^9^. The present study analyzed prosthesis use among older adults who had lost 13 or more teeth and who require fixed or removable partial dentures, which in turn are scarcely available throughout the country^9^. This reflects, in part, a historical pattern of care models centered on the individual and on low-complexity procedures^19^. Furthermore, the strong influence of structural ageism must also be considered, as its manifestations in health-especially oral health-are evident^20^.

Older black individuals were more affected by tooth loss. This result may be related to the persistence of socioeconomic inequalities resulting from historical processes of exploitation of this population^11^ ^,^ ^21^, leading to reduced availability of economic and structural resources and difficulties in accessing dental services^11^. The subjective nature of self-assessment in oral health is also noteworthy, as it negatively impacts the perception of oral needs and problems, reflecting a set of historical, social, and cultural experiences within the black population^11^ ^,^ ^22^ ^,^ ^23^.

Another relevant aspect concerns the way dental care has been offered to the black population. Evidence indicates that these individuals are more likely to undergo emergency procedures or tooth extractions, to the detriment of conservative and restorative approaches^24^. This difference may be related to the influence of race/skin color on clinical decision-making, revealing racism in professional conduct^22^. Structural racism is also manifested in health practices and decisions^22^ ^,^ ^24^, contributing to the persistence of inequalities in dental care^25^.

The intersectional analysis of race/color and gender in oral health showed that older black women present the worst indicators of tooth loss, while black men are the least rehabilitated. These differences reflect the historical burden of racial and gender inequalities, associated with income, double work shifts, and violence against black women^26^. These factors operate as structural social determinants of health, influencing cumulative exposure to oral disease throughout the life course and timely, effective access to dental services.

Although women experience greater tooth loss, they tend to seek dental services more often and use dental prostheses more frequently than men^23^ ^,^ ^27^, possibly due to greater self-perception and attention to health^23^ ^,^ ^28^. However, this does not translate into better oral health conditions^29^, as evidenced in the present study, in line with the literature^28^ ^,^ ^30^ ^,^ ^31^. This scenario is exacerbated when race/color and gender markers overlap, resulting in greater vulnerability among older black women, similarly to another study^6^ hat also identified greater tooth loss among black women.

Among men, the lower demand for services may be related to time constraints, fear of diagnosis, and cultural patterns of masculinity that discourage self-care^23^. These gender differences highlight the structural limits of care both in illness and in opportunities for treatment and rehabilitation. From an intersectional perspective, black men perceive health needs based on socio-historical-cultural experiences, placing them at a disadvantage compared to white men in access to prosthetic rehabilitation. In addition, race influences the dentist’s choice of treatment^32^. For example, another study^29^ found that black individuals with decayed teeth were 50% less likely to be referred for prosthetic treatment.

The regional inequalities identified demonstrate that place of residence exerts a strong influence on oral health conditions in old age. The Southeast and South regions, as well as living in state capitals, showed the best oral health conditions^7^ ^,^ ^8^ ^,^ ^19^. This difference can be explained, in part, by the unequal distribution of dental professionals across the country, since although the total number of dentists is high, their concentration is greater in the more developed regions and in the private sector^33^. This disparity creates significant barriers to access to preventive and rehabilitative care, contributing to the persistence of territorial inequities and greater vulnerability among older adults living outside major urban centers.

Furthermore, distinct socioeconomic and demographic factors across regions influence the aging process and the use of dental services, reinforcing unequal care^34^ ^,^ ^35^ ^,^ ^36^. From the perspective of dental prosthesis provision, there has been unequal growth in the number of Regional Dental Prosthesis Laboratories (LRPD)^37^ and variation in prosthesis production among Brazil’s regions^21^, revealing regional disparities in oral health policy.

Thus, even with the expansion of healthcare networks in the country, gaps persist in access to dental services for certain population groups^36^, especially the black population. One study identified the presence of racial inequality in the provision of complete dentures in the country, since, even in municipalities with a higher proportion of the black population, a greater supply of oral health teams and Dental Specialty Centers (CEOs), it was observed that the provision of dental prostheses was greater for the white population^38^.

From an intersectional regional perspective, the national pattern of greater tooth loss among black women and lower denture use among black men is maintained; however, regional particularities become evident in the territorial intersectional analysis.

In the North Region, despite the worst average oral health indicators, no significant inequalities were observed according to gender-race. This finding may reflect a context of high structural vulnerability, in which geographic barriers, lower availability of dental services, and historical territorial inequalities broadly affect the elderly population, attenuating differences in outcomes among the analyzed groups. While the Southeast Region showed no gender-race inequalities in the prevalence of edentulism and denture use, the South Region showed no inequality in edentulism. In contrast, in the Central-West Region, white men stand out as particularly privileged regarding lower tooth loss and better functional dentition compared with all other gender-race categories, revealing more pronounced inequalities than in other regions. This scenario reflects the persistence of historical structural inequalities and highlights the need for regionalized policies and strategies that consider the social, economic, and cultural specificities of each territory.

Analyzing these demands from individual, contextual, and racial perspectives is essential for planning fairer public policies aimed at equity in oral health, ensuring priority for historically vulnerable groups^11^ ^,^ ^12^ ^,^ ^19^. However, considering the history of high tooth loss and low rehabilitation among older Brazilian adults, it is likely that significant improvements in this situation will occur only with the newer generations, who tend to age with better oral health conditions.

This study highlights the importance of incorporating an intersectional perspective into oral health policies aimed at the elderly population, and that these policies dialogues with the Comprehensive Health Policy for the Black Population^39^, in order to combat systemic racism in the field of oral health and all forms of discrimination in any space of life^40^. It is necessary that public policies with affirmative action and educational programs aimed at benefiting black women be maintained and rethought, in order to ensure universal and equitable access, in accordance with the principles of the SUS^6^ ^,^ ^36^. The consideration of gender, race, and territory markers in the planning, monitoring, and evaluation of interventions can contribute to reducing inequalities and strengthening the SUS.

Among the limitations of this study is the non-inclusion of the 60-64 years old population in SB Brasil, excluding part of the elderly black population, given that their life expectancy is lower than that of the white population. Another limitation was the analysis of the black category, which may obscure internal heterogeneities between black and brown individuals, as well as the analysis of functional dentition without considering the occlusal pairs and without discriminating the use of different types of dental prostheses.

Despite advances in oral health among elderly Brazilians, inequalities persist, marked by the intersection of gender, race, and territory, with a more severe impact on black women and individuals residing in historically disadvantaged regions. The maintenance of these inequities reveals the limitations of universal policies and reinforces the need for affirmative action strategies within the Brazilian Unified Health System (SUS), which consider the sociocultural and territorial specificities of different population groups.

## References

[1] Ferreira RC, Vargas AMD, Moura RNV, Fonseca MLV, Gomes VE, Pinheiro EL (2025). Caries and edentulism trends among Brazilian older adults: a comparative analysis of 2003, 2010, and 2023 surveys. Braz Oral Res.

[2] Farias BO, Araújo LF, Tavares NHC, Oliveira BBR, Vasconcelos MSL, Machado SP (2025). Interseccionalidade gênero, raça/cor da pele e perda dentária em adultos: estudo transversal com dados da Pesquisa Nacional de Saúde 2019. Epidemiol Serv Saude.

[3] Oliveira EJP, Alves LC, Santos JLF, Duarte YAO, Andrade FB (2020). Edentulism and all-cause mortality among Brazilian older adults: 11-years follow-up. Braz Oral Res.

[4] Lipsky MS, Singh T, Zakeri G, Hung M (2024). Oral health and older adults: a narrative review. Dent J (Basel).

[5] Kalache A, Lima KC, Louvison M, Silva VL (2023). Envelhecimento, velhices e interseccionalidades. Rev Bras Geriatr Gerontol.

[6] Terra e Souza LH, Diaz-Quijano FA, Azevedo Barros MB, Lima MG (2022). Race (black-white) and sex inequalities in tooth loss: a population-based study. PLoS One.

[7] Cericato GO, Agostini BA, Costa FDS, Thomson WM, Demarco FF (2021). Rural-urban differences in oral health among older people in Southern Brazil. Braz Oral Res.

[8] Schroeder FMM, Mendoza-Sassi RA, Meucci RD (2020). Condição de saúde bucal e utilização de serviços odontológicos entre idosos em área rural no sul do Brasil. Cien Saude Colet.

[9] Teixeira AKM, Souza PRB (2025). Evolution of socioeconomic inequalities in oral health and use of dental services in adult population of Brazil between 2013 and 2019. Cad Saude Publica.

[10] Miranda LP, Oliveira TL, Queiroz PSF, Oliveira PSD, Fagundes LS, Rodrigues JF (2020). Saúde bucal e acesso aos serviços odontológicos em idosos quilombolas: um estudo de base populacional. Rev Bras Geriatr Gerontol.

[11] Menegazzo GR, Cunha AR, Fagundes MLB, Amaral OL, Giordan JMA, Hilgert JB (2023). Pathways that explain racial differences on edentulism among older adults: 2019 Brazil National Health Survey. Braz Oral Res.

[12] Oliveira ECT, Louvison MCP, Oliveira Duarte YA, Andrade FB (2025). Socioeconomic inequalities related to perceived difficulty in accessing health services among older adults: a cross-sectional analysis of SABE Study Data. PLoS One.

[13] Brasil. Ministério da Saúde (2022). SB Brasil 2020: Pesquisa Nacional de Saúde Bucal: projeto técnico.

[14] Brasil. Ministério da Saúde. Secretaria de Atenção à Saúde (2009). SB Brasil 2010: Pesquisa Nacional de Saúde Bucal: resultados principais.

[15] Roncalli AG, Silva NN, Nascimento AC, Freitas CHSM, Casotti E, Peres KG (2012). Aspectos metodológicos do Projeto SBBrasil 2010 de interesse para inquéritos nacionais de saúde. Cad Saude Publica.

[16] World Health Organization (2013). Springer Topics in Signal Processing.

[17] Brasil (2010). Lei nº 12.288, de 20 de julho de 2010. Institui o Estatuto da Igualdade Racial, altera as Leis n^os^ 7.716, de 5 de janeiro de 1989, 9.029, de 13 de abril de 1995, 7.347, de 24 de julho de 1985, e 10.778, de 24 de novembro de 2003.

[18] Bastos TF, Medina LPB, Sousa NFS, Lima MG, Malta DC, Barros MBA (2019). Income inequalities in oral health and access to dental services in the Brazilian population: National Health Survey, 2013. Rev Bras Epidemiol.

[19] Veiga Pessoa DM, Roncalli AG, Lima KC (2016). Economic and sociodemographic inequalities in complete denture need among older Brazilian adults: a cross-sectional population-based study. BMC Oral Health.

[20] Menezes EEG, Botacin LA, Leles CR, Marchini L, Nogueira TE (2023). Idadismo contra pessoas idosas e fatores associados: um estudo transversal com estudantes de Odontologia. Rev Odontol UNESP.

[21] Aguiar VR, Celeste RK (2015). Necessidade e alocação de laboratórios regionais de prótese dentária no Brasil: um estudo exploratório. Cienc Saúde Colet.

[22] Candido LC, Finkler M, Bastos JL, Freitas SFT (2019). Conflitos com o paciente, cor/raça e concepções de estudantes de Odontologia: uma análise com graduandos no Sul do Brasil. Physis.

[23] Cunha RO, Correia PSM, Nogueira MC, Leite ICG (2025). Iniquidades raciais em saúde bucal na população brasileira: uma análise da Pesquisa Nacional de Saúde de 2019. Cien Saude Colet.

[24] Patel N, Patel S, Cotti E, Bardini G, Mannocci F (2019). Unconscious racial bias may affect dentists’ clinical decisions on tooth restorability: a randomized clinical trial. JDR Clin Trans Res.

[25] Raskin SE, Thakkar-Samtani M, Santoro M, Fleming EB, Heaton LJ, Tranby EP (2023). Discrimination and dignity experiences in prior oral care visits predict racialized oral health inequities among nationally representative US adults. J Racial Ethn Health Disparities.

[26] Marcacine PR, Castro SS, Castro SS, Meirelles MCCC, Haas VJ, Walsh IAP (2019). Qualidade de vida, fatores sociodemográficos e ocupacionais de mulheres trabalhadoras. Cien Saude Colet.

[27] Pinheiro RS, Viacava F, Travassos C, Brito AS (2002). Gênero, morbidade, acesso e utilização de serviços de saúde no Brasil. Cien Saúde Colet.

[28] Bortoli FR, Moreira MA, Moretti-Pires RO, Botazzo C, Kovaleski DF (2017). Percepção da saúde bucal em mulheres com perdas dentárias extensas. Saude Soc.

[29] Susin C, Oppermann RV, Haugejorden O, Albandar JM (2005). Tooth loss and associated risk indicators in an adult urban population from south Brazil. Acta Odontol Scand.

[30] Peres MA, Barbato PR, Reis SCGB, Freitas CHSDM, Antunes JLF (2013). Perdas dentárias no Brasil: análise da Pesquisa Nacional de Saúde Bucal 2010. Rev Saúde Pública.

[31] Bomfim RA, Schneider IJC, de Andrade FB, Lima-Costa MF, Corrêa VP, Frazão P (2021). Racial inequities in tooth loss among older Brazilian adults: a decomposition analysis. Community Dent Oral Epidemiol.

[32] Chisini LA, Noronha TG, Ramos EC, Santos-Junior RB, Sampaio KH, Faria-e-Silva AL (2019). Does the skin color of patients influence the treatment decision-making of dentists? A randomized questionnaire-based study. Clin Oral Investig.

[33] Cascaes AM, Dotto L, Bomfim RA (2018). Tendências da força de trabalho de cirurgiões-dentistas no Brasil, no período de 2007 a 2014: estudo de séries temporais com dados do Cadastro Nacional de Estabelecimentos de Saúde. Epidemiol Serv Saude.

[34] Bomfim RA, Cascaes AM, Oliveira C (2021). Multimorbidity and tooth loss: the Brazilian National Health Survey, 201. BMC Public Health.

[35] Andrade FB, Antunes JLF, Andrade FCD, Lima-Costa MFF, Macinko J (2020). Education-related inequalities in dental services use among older adults in 23 countries. J Dent Res.

[36] Galvão MHR, Souza ACO, Morais HGF, Roncalli AG (2022). Desigualdades no perfil de utilização de serviços odontológicos no Brasil. Ciên Saúde Colet.

[37] Chaves SCL, Lima AMFS, Rossi TA (2025). Recomendações e destaques: aprimorando a oferta de próteses dentárias no SUS.

[38] Bomfim RA, Lucena EHG, Cavalcanti YW, Celeste RK (2023). Racial inequality in complete dental prosthesis delivered: can public services reduce inequities?. Clin Oral Investig.

[39] Brasil. Ministério da Saúde. Secretaria de Gestão Estratégica e Participativa (2017). Política Nacional de Saúde Integral da População Negra.

[40] Santos MPA, Bastos JL (2024). Ethos antirracista em saúde bucal coletiva como imperioso à vida. Cien Saúde Colet.

