# Supplementary material for: Tooth loss and rehabilitation in the elderly: an intersectional analysis of gender, race, and territory
Source: Rev Bras Epidemiol. 2026 Jul 24;29(Suppl 1):e260020supl1. doi: 10.1590/1980-549720260020.supl.1 (PMC13403694; doi:10.1590/1980-549720260020.supl.1)
Supplement: Translation

# Translation: Perda dentária e reabilitação na pessoa idosa: análise interseccional de gênero-raça-território

**DOI:** 10.1590/1980-549720260020.supl.1.2

**Authors:** Ana Karine Macedo Teixeira, Raul Anderson Domingues Alves da Silva, Alice Castro Guedes Mendonça, Kenio Costa de Lima

**Affiliations:** I Universidade Federal do Ceará - Fortaleza (CE), Brasil.; II Universidade Christus - Fortaleza (CE), Brasil.; III Universidade Federal do Rio Grande do Norte - Natal (RN), Brasil.

**Keywords:** Enquadramento interseccional, Perda de dente, Saúde bucal, Desigualdades de saúde

## Abstract

**Objetivo:**

Avaliar a perda dentária, funcionalidade dentária e reabilitação em pessoas idosas em 2010 e 2023 e sua interseção entre gênero-raça-território.

**Métodos:**

Utilizou-se amostra de idosos da Pesquisa Nacional de Saúde Bucal (SB Brasil) 2023 (n=9.745) e 2010 (n=7.619). Os desfechos foram: a perda dentária, a funcionalidade dentária e a reabilitação oral da população idosa. Os dados demográficos (sexo, raça, idade e regiões brasileiras) foram utilizados como variáveis independentes. Realizou-se regressão de Poisson para cada variável de saúde bucal com base em um modelo de interação entre raça/cor e gênero, estratificada por região.

**Resultados:**

A condição de saúde bucal da população idosa melhorou entre 2010 e 2023, com redução do edentulismo (de 53,4 para 36%), e aumento da dentição funcional (de 13,2 para 28,6%) e da dentição anterior completa (de 12,4 para 21,2%). O uso de prótese nas pessoas idosas que apresentaram menos de 20 dentes não melhorou no período avaliado. Na análise interseccional, mulheres negras apresentaram maior prevalência de edentulismo (RP=1,16; IC 1,04-1,29), menor dentição funcional (RP=0,74; IC 0,66-0,83) e menor dentição anterior (RP=0,85; IC 0,78-0,93) em comparação ao homem branco, enquanto o homem negro é o menos reabilitado (RP=0,83; IC 0,78-0,90). As desigualdades de gênero e raça variaram entre as regiões. A região Norte, apesar das piores condições de saúde bucal da população idosa, não apresentou desigualdade na dentição funcional e no edentulismo.

**Conclusão:**

Apesar dos avanços na saúde bucal dos idosos brasileiros, persistem desigualdades marcadas pela interseção entre gênero, raça e território, com impacto mais severo sobre mulheres negras.

## INTRODUÇÃO

A persistência de doenças bucais destaca a existência de profundas iniquidades e desigualdades econômicas no mundo^1^ ^,^ ^2^. No Brasil, a transição demográfica resultou no rápido envelhecimento populacional^1^ ^,^ ^3^, tornando a saúde das pessoas idosas, incluindo a saúde bucal, um aspecto relevante para as políticas públicas^1^.

A perda dentária, especialmente o edentulismo, é consequência direta da exposição prolongada a doenças bucais, refletindo o acúmulo de experiências de saúde ao longo da vida, e considerada marcador de saúde bucal em indivíduos mais velhos^1^ ^,^ ^3^. O envelhecimento é um fator de risco significativo, com pico de perda dentária grave ocorrendo em pessoas idosas^1^ ^,^ ^4^. Embora o Brasil tenha registrado redução na prevalência de edentulismo entre 65-74 anos^1^, aproximadamente 35% dessa população ainda é edêntula, superando a média global estimada^1^. O edentulismo está associado a consequências graves, incluindo comprometimento da mastigação, nutrição, qualidade de vida e risco maior de mortalidade por todas as causas, mesmo após ajuste para fatores socioeconômicos e de saúde^1^ ^,^ ^3^ ^,^ ^4^.

Para compreender a distribuição desigual da perda dentária, é essencial considerar os determinantes sociais^1^, e a lente da interseccionalidade oferece a estrutura teórica necessária para explorar a complexidade da saúde em populações vulnerabilizadas^2^ ^,^ ^5^. O conceito de interseccionalidade, enraizado no movimento negro feminista^5^, propõe que os eixos de opressão social, como gênero, raça/cor da pele e classe social, interagem e se sobrepõem, resultando em diferentes níveis de vulnerabilidade e exclusão em saúde^2^ ^,^ ^5^. A aplicação dessa perspectiva é primordial para garantir o direito ao envelhecimento digno e para que políticas públicas reconheçam as iniquidades que impedem muitos grupos de alcançarem os 60 anos^5^.

As evidências no Brasil demonstram que reduções na perda dentária não ocorreram de maneira uniforme, sendo mais expressivas entre indivíduos brancos e aqueles com maior escolaridade^1^. O racismo estrutural e o sexismo são reconhecidos como mecanismos sociais que impulsionam as iniquidades^1^ ^,^ ^6^. Na perspectiva da interseccionalidade, essas evidências não podem ser compreendidas isoladamente, mas sim com a sobreposição dos eixos de opressão social na trajetória de vida dessas pessoas que levaram alguns grupos a ter mais acesso a cuidados em saúde bucal do que outros.

Em particular, a interseccionalidade entre raça e gênero revela uma vulnerabilidade exacerbada^6^. Embora o gênero possa ser um fator de grande importância, o impacto de mecanismos sociais como o racismo e o sexismo pode ser mais pronunciado na saúde bucal feminina^2^.

Outro aspecto que interage com o desfecho da perda dentária é o território. Evidências mostram que morar na zona rural tem impacto negativo na qualidade da saúde bucal de idosos brasileiros^7^ ^,^ ^8^, mas não se conhece como a interseção de raça-gênero impacta a saúde bucal de idosos em diferentes regiões do país.

Nesse contexto, é importante reconhecer que, apesar de avanços da Política de Saúde Bucal no país, as desigualdades persistem^9^. A condição de saúde bucal da população negra, incluindo a população quilombola^10^, pode, em parte, ser explicada pelas limitações de acesso aos serviços de saúde, em decorrência da ausência de transporte público adequado, insegurança ou recursos financeiros para tal^11^. Quando se trata da pessoa idosa, essas questões são ainda mais relevantes, por se tratar de uma população que tradicionalmente encontra dificuldades de deslocamento e mobilidade^12^.

Portanto, monitorar a perda e funcionalidade dentárias e a reabilitação em pessoas idosas brasileiras, ampliando a compreensão da sobreposição de desigualdades sob a lente da interseccionalidade nas regiões brasileiras, é fundamental para identificar os grupos mais vulnerabilizados e contribuir para o desenvolvimento de intervenções e políticas de saúde que sejam mais equitativas e capazes de superar barreiras sociais estruturais que perpetuam as iniquidades em saúde bucal^1^ ^,^ ^2^. Assim, o objetivo do estudo foi avaliar a perda dentária, a funcionalidade dentária e a reabilitação protética em pessoas idosas em 2010 e 2023 e sua interseção gênero-raça-território.

## MÉTODOS

Este estudo transversal, de base populacional, foi realizado utilizando dados secundários da Pesquisa Nacional de Saúde Bucal (SB Brasil) 2023 e 2010, disponibilizados pelo Ministério da Saúde. Em 2010, a amostragem foi composta por 32 domínios, incluindo capitais e municípios do interior do país, com participantes selecionados por meio de amostragem por conglomerados. Em 2023, a pesquisa abrangeu 53 domínios, incluindo estados e capitais. A amostragem permitiu que estimativas fossem feitas para as cinco regiões brasileiras nas duas edições. O detalhamento dos aspectos metodológicos do SB Brasil está disponível em outras publicações^13^ ^,^ ^14^ ^,^ ^15^.

A coleta de dados envolveu entrevistas e exames bucais realizados em domicílio por profissionais de saúde bucal do Sistema Único de Saúde (SUS), treinados e calibrados para garantir a consistência dos exames. O Comitê Nacional de Ética em Pesquisa aprovou ambas as pesquisas (2010: protocolo n.º 15.498; 2023: protocolo n.º 4.823.054), e os participantes assinaram um termo de consentimento informado.

Para este estudo, utilizou-se a amostra de pessoas idosas de 65 a 74 anos que participaram das duas edições, totalizando 7.619 em 2010 e 9.745 idosos em 2023.

Foram utilizados seis desfechos para medir a condição bucal de acordo com a perda e funcionalidade dentária e a reabilitação protética da população idosa, considerando os critérios de exames da Organização Mundial da Saúde (OMS) e dos manuais técnicos do SB Brasil^13^ ^,^ ^14^ ^,^ ^16^: 1) Índice CPOD (número médio de dentes permanentes com experiência de cárie - dentes cariados, perdidos e restaurados); 2) Média de dentes perdidos (número médio de dentes permanentes perdidos que compõe o CPOD); 3) Edentulismo (sim/não) - perda de todos os dentes, incluindo dentes perdidos por outras razões que não a cárie dentária -; 4) Dentição funcional (sim/não) - presença de 20 ou mais dentes na boca, sem considerar a presença de pares em oclusão -; 5) Dentição completa anterior (sim/não) - presença de todos os dentes do segundo e do quinto sextantes -; 6) Uso de prótese (sim/não) - uso de algum tipo de prótese dentária por pessoas idosas que possuem menos de 20 dentes.

Como variáveis independentes, utilizaram-se as variáveis demográficas: sexo (masculino/feminino), raça/cor (brancos/negros), idade (65-69 anos/70-74 anos), regiões brasileiras (Norte, Nordeste, Sudeste, Centro-Oeste, Sul) e tipo de cidade (capital/interior).

A análise interseccional de gênero-raça foi realizada apenas na amostra de 2023. Para a definição de gênero utilizou-se a variável “sexo feminino” para mulher e “masculino” para homem. Na análise racial, considerou-se a população negra, com base na autodeclaração como pardas e pretas, excluindo-se da análise a população indígena, amarela ou não informados (amostra final 2023 = 9.414). A categorização racial vai de acordo com o referencial da epidemiologia em saúde coletiva e com as diretrizes das políticas públicas brasileiras^17^.

A análise foi realizada no software Stata 13.0, utilizando o módulo survey para considerar o desenho amostral e os pesos derivados do processo de amostragem. Inicialmente, obteve-se a análise descritiva das variáveis demográficas e de saúde bucal (perda e funcionalidade dentária e reabilitação protética) para os anos de 2010 e 2023, apresentando as estimativas de prevalências e seus respectivos intervalos de confiança (95%). Na sequência, testou-se a hipótese de associação entre as variáveis de saúde bucal e as variáveis demográficas pelo teste qui-quadrado de Pearson, com nível de significância de 5%. Para análise interseccional, utilizou-se a regressão de Poisson para cada desfecho de saúde bucal (edentulismo, dentição funcional, dentição anterior e uso de prótese), um modelo de interação entre as variáveis raça/cor e gênero. Por fim, estratificou-se a análise interseccional para cada variável dependente por região. Para todas as análises utilizou-se Razão de Prevalência (RP) com Intervalo de Confiança (IC) de 95%.

## Declaração de Disponibilidade de Dados:

Todo o conjunto de dados que dá suporte aos resultados deste estudo é proveniente da Pesquisa Nacional de Saúde Bucal (SBBrasil 2023) e está disponível mediante solicitação ao Ministério da Saúde do Brasil. O conjunto de dados não está publicamente disponível, uma vez que contém informações sensíveis que podem comprometer a privacidade e a confidencialidade dos participantes da pesquisa, sendo seu acesso condicionado à autorização do órgão responsável.

## RESULTADOS

A caracterização da amostra de pessoas idosas participantes do SB Brasil nos anos de 2010 e 2023 manteve o padrão, com maioria composta pelo sexo feminino, de raça/cor branca, na idade de 65 a 69 anos, residente no Sudeste e no interior do país. Entretanto, observou-se aumento no número de pessoas idosas negras, residentes no Nordeste e no interior entre os dois levantamentos (Tabela 1).

**Tabela 1.** Distribuição das variáveis demográficas de pessoas idosas. SB Brasil 2010 e 2023.

	2010 % (IC)	2023 % (IC)
Sexo		
Feminino	62,2 (58,7-65,6)	60,5 (58,2-62,7)
Masculino	37,8 (34,4-41,3)	39,5 (37,3-41,8)
Raça		
Branco	55,8 (51,5-60,0)	50,3 (46,5-54,1)
Negro	44,2 (40,0-48,5)	49,7 (45,9-53,5)
Idade (anos)		
65 a 69	54,9 (51,5-58,2)	56,1 (52,4-59,8)
70 a 74	45,1 (41,8-48,5)	43,9 (40,2-47,6)
Região		
Norte	5,1 (3,5-7,5)	5,6 (4,8-6,5)
Nordeste	9,8 (8,0-11,8)	24,1 (21,4-27,0)
Sudeste	62,8 (57,3-68,1)	46,9 (42,6-51,2)
Centro-Oeste	6,1 (4,8-7,6)	6,7 (5,3-8,6)
Sul	16,2 (13,2-19,7)	16,7 (14,3-19,3)
Tipo de cidade		
Capital	32,1 (25,3-39,8)	23,8 (21,4-26,3)
Interior	67,7 (60,2-74,7)	76,2 (73,7-78,6)

Quanto à condição de saúde bucal (Tabela 2), o CPOD reduziu de 27,5 (DP=0,25) em 2010 para 23,5 (DP=0,31) em 2023, com redução na média de quase cinco dentes perdidos. O edentulismo, por sua vez, reduziu de 53,4 para 36,5%, e houve melhora na funcionalidade dentária, considerando a dentição funcional e a dentição anterior completa, que alcançaram prevalência de 28,6 e 21,2% em 2023, respectivamente. Entretanto, a prevalência do uso de prótese nos idosos com menos de 20 dentes manteve valores aproximados entre 2010 (84,2%) e 2023 (85,3%).

**Tabela 2.** Prevalência das variáveis de saúde bucal (perda dentária, funcionalidade e reabilitação) de idosos. SB Brasil 2010 e 2023.

	2010	2023
	Média (DP)	Média (DP)
CPOD	27,5 (DP=0,25)	23,5 (DP=0,31)
Média de dentes perdidos	25,2 (DP=0,38)	19,8 (DP=0,49)
	% (IC)	% (IC)
Edentulismo	53,4 (49,4-58,0)	36,5 (33,4-39,6)
Dentição funcional	13,2 (11,0-15,8)	28,6 (25,5-32,0)
Dentição anterior completa	12,4 (10,0-15,3)	21,2 (18,9-23,8)
Uso de prótese em idosos com menos de 20 dentes	84,2 (81,3-86,7)	85,3 (82,5-87,6)

As mulheres apresentaram maior edentulismo (38,5%), menor funcionalidade dentária (25%) e menor dentição anterior completa (19,4%) do que os homens, mas também maior prevalência no uso de prótese (87,8%). A faixa etária de 70 a 74 anos possui maior perda dentária, mas não demonstrou diferença na reabilitação protética em relação à faixa etária de 64 a 69 anos. As pessoas idosas negras contabilizaram mais dentes perdidos do que as pessoas brancas, entretanto, pessoas negras (81,6%) foram menos reabilitadas do que as brancas (89,3%). A região Sudeste possui mais idosos com 20 ou mais dentes (35,3%) e com a dentição anterior completa (25,7%), enquanto a região Sul foi a que apresentou maior prevalência de pessoas idosas que usam algum tipo de prótese (92,2%). Ainda, idosos que moram no interior (37,8%) têm maior edentulismo do que os que residem na capital (32,4%) (Tabela 3).

**Tabela 3.** Prevalência das variáveis de saúde bucal (perda dentária, funcionalidade e reabilitação) segundo as características demográficas. SB Brasil 2023.

	Edentulismo % (IC)	Dentição funcional % (IC)	Dentição anterior completa % (IC)	Uso de prótese % (IC)
Sexo				
Masculino	33,4 (30,0-37,1)	34,1 (30,1-38,4)	24,0 (20,7-27,7)	80,8 (76,7-84,3)
Feminino	38,5 (34,4-42,7)*	25,0 (21,6-28,8)**	19,4 (16,6-22,5)*	87,8 (85,1-90,1)**
Idade (anos)				
65 a 69	31,5 (27,8-35,4)	32,5 (28,7-36-6)	23,7 (20,8-26,8)	86,1 (82,7-89,0)
70 a 74	42,8 (38,8-47,0)**	23,7 (19,7-28,6)**	18,0 (14,8-21,8)*	84,3 (80,8-87,2)
Raça/cor				
Branco	34,5 (30,0-39,6)	33,5 (28,6-38,7)	25,0 (21,4-29,1)	89,3 (66,9-74,8)
Negro	38,6 (35,5-41,7)	23,9 (21,0-27,1)**	17,6 (15,4-20,1)**	81,6 (77,5-85,1)**
Região				
Norte	39,9 (35,3-44,8)	18,1 (13,7-23,6)	14,0 (9,8-19,6)	82,4 (77,4-86,5)
Nordeste	37,6 (34,0-41,4)	21,9 (18,3-26,1)	18,7 (15,8-22,0)	78,0 (72,4-82,7)
Sudeste	35,2 (29,6-41,4)	35,3 (29,5-41,6)**	25,7 (21,4-30,6)**	86,8 (81,3-90,8)
Centro-Oeste	40,5 (35,4-45,7)	23,8 (19,9-28,1)	17,4 (14,5-20,6)	88,6 (83,8-92,1)
Sul	35,5 (30,2-41,3)	25,0 (20,6-29,9)	16,1 (13,4-19,2)	92,2 (88,9-94,6)**
Tipo de cidade				
Capital	32,4 (29,4-35,4)	30,5 (28,0-33,1)	23,6 (21,5-25,8)	85,6 (83,3-87,7)
Interior	37,8 (33,9-41,8)*	28,0 (24,0-32,4)	20,5 (17,5-23,8)	85,1 (81,6-88,1)

*p<0,05; **p<0,001.

Na análise interseccional de gênero-raça (Tabela 4), observou-se que a idosa negra apresentou a pior condição bucal para todos os indicadores de perda dentária. A prevalência de edentulismo na mulher negra foi 1,16 (IC 1,04-1,29) vezes maior que no homem branco, e este apresentou a melhor condição de dentição funcional, comparada com todas as outras categorias de gênero-raça, com maior desigualdade para a mulher negra (RP=0,74, IC 0,66-0,83). Já na dentição completa anterior, o homem branco se diferenciou apenas da mulher negra, que apresentou menor proporção de dentes na região anterior (RP=0,85, IC 0,78-0,93). No uso de prótese, a mulher branca apresentou melhor condição, comparada com todas as outras categorias, inclusive com a mulher negra (RP=0,91, IC 0,86-0,96), e o homem negro foi o menos reabilitado (RP=0,83, IC 0,78-0,90).

**Tabela 4.** Análise interseccional de gênero e raça das variáveis de saúde bucal (perda dentária, funcionalidade e reabilitação). SB Brasil 2023.

Gênero-raça/cor	n	Edentulismo		Dentição funcional		Dentição anterior completa		Uso de prótese	
		% (IC)	RP (IC)	% (IC)	RP (IC)	% (IC)	RP (IC)	% (IC)	RP (IC)
Homem branco	1.322	31,1 (25,7-37,1)	1	41,0 (34,2-48,1)	1	28,4 (22,8-34,8)	1	84,3 (78,0-89,0)	0,91* (0,85-0,98)
Mulher branca	2.277	36,8 (31,1-42,9)	1,09 (0,97-1,21)	28,5 (23,6-33,9)	0,83** (0,74-0,92)	22,8 (18,5-27,7)	0,93 (0,84-1,02)	92,0 (88,4-94,5)	1
Homem negro	2.276	35,5 (31,6-39,6)	1,07 (0,96-1,18)	28,1 (23,4-33,3)	0,82* (0,70-0,95)	20,2 (16,3-24,8)	0,89* (0,81-1,00)	77,2 (71,7-81,9)	0,83** (0,78-0,90)
Mulher negra	3.539	40,6 (36,3-45,0)	1,16* (1,04-1,29)	21,2 (17,5-25,5)	0,74** (0,66-0,83)	16,0 (13,1-19,3)	0,85** (0,78-0,93)	84,2 (79,9-87,7)	0,91* (0,86-0,96)

*p<0,05; **p<0,001.

Ao acrescentar a região na análise interseccional de gênero-raça (Tabela 5), observaram-se comportamentos diferentes para cada região. A região Norte, apesar de possuir os piores indicadores de perda dentária do país, não apresentou desigualdades de gênero-raça para edentulismo, dentição funcional e dentição completa anterior. Já na reabilitação, verificou-se que a mulher branca utiliza mais prótese dentária, enquanto o idoso negro da região Norte possui menor chance de ser reabilitado (RP=0,89, IC 0,83-0,94).

**Tabela 5.** Análise interseccional de gênero-raça-região com as variáveis de saúde bucal (perda dentária, funcionalidade e reabilitação). SB Brasil 2023.

Região	Gênero-raça/cor (N)	Edentulismo		Dentição funcional		Dentição anterior completa		Uso de prótese	
		% (IC)	RP (IC)	% (IC)	RP (IC)	% (IC)	RP (IC)	% (IC)	RP (IC)
Norte (n=2.165)	Homem branco (165)	42,1 (29,5-55,9)	1	17,2 (8,4-31,9)	1	10,5 (5,6-18,8)	1	83,9 (74,6-90,2)	0,89* (0,81-0,98)
	Mulher branca (258)	41,7 (28,0-56,8)	0,99 (0,67-1,46)	17,6 (11,4-26,0)	1,01 (0,87-1,15)	14,0 (8,1-23,1)	1,04 (0,96-1,15)	94,2 (86,4-97,7)	1
	Homem negro (759)	38,5 (32,1-45,3)	0,94 (0,72-1,22)	18,6 (14,3-23,8)	1,01 (0,88-1,17)	14,6 (10,3-20,3)	1,04 (0,95-1,15)	76,8 (70,5-82,2)	0,81** (0,76-0,87)
	Mulher negra (983)	40,2 (33,7-47,0)	0,96 (0,76-1,22)	18,0 (10,8-28,5)	1,01 (0,85-1,19)	14,3 (7,4-25,7)	1,04 (0,92-1,18)	84,0 (78,2-88,5)	0,89* (0,83-0,94)
Nordeste (n=3.135)	Homem branco (263)	28,3 (19,4-39,4)	1	32,7 (21,1-46,8)	1	27,4 (16,9-41,1)	1	82,7 (72,4-89,8)	0,98 (0,86-1,14)
	Mulher branca (573)	45,2 (37,2-53,4)	1,31* (1,06-1,61)	20,3 (13,6-29,0)	0,89 (0,68-1,05)	22,2 (15,5-30,8)	0,93 (0,76-1,15)	83,9 (76,7-89,2)	1
	Homem negro (850)	35,4 (29,4-41,8)	1,10 (0,95-1,29)	28,3 (20,6-37,4)	0,93 (0,75-1,16)	21,4 (14,7-30,0)	0,93 (0,75-1,13)	70,9 (61,4-78,9)	0,84* (0,74-0,97)
	Mulher negra (983)	39,2 (34,1-44,6)	1,18* (1,01-1,38)	15,9 (12,7-19,7)	0,80* (0,66-0,96)	13,6 (10,8-17,1)	0,84* (0,71-0,99)	79,2 (71,9-85,0)	0,94 (0,85-1,05)
Sudeste (n=1.418)	Homem branco (280)	31,3 (23,1-41,0)	1	47,8 (37,5-58,4)	1	34,4 (26,0-43,9)	1	81,5 (69,8-89,4)	0,89 (0,79-1,03)
	Mulher branca (432)	34,7 (25,0-45,9)	1,05 (0,88-1,27)	34,2 (25,8-43,9)	0,79* (0,65-0,96)	26,6 (19,0-35,7)	0,89 (0,74-1,07)	90,8 (83,3-95,1)	1
	Homem negro (239)	31,8 (24,9-39,7)	1,01 (0,85-1,19)	34,6 (25,8-44,7)	0,79 (0,60-1,05)	24,4 (17,1-33,5)	0,87 (0,71-1,06)	85,2 (73,4-92,3)	0,94 (0,83-1,06)
	Mulher negra (467)	40,8 (32,8-49,4)	1,16 (0,96-1,39)	25,9 (18,5-35,0)	0,70** (0,57-0,85)	18,7 (13,2-25,8)	0,81** (0,70-0,93)	86,3 (77,4-92,0)	0,95 (0,86-1,05)
Centro-Oeste (n=1.299)	Homem branco (210)	22,3 (14,1-33,4)	1	42,7 (30,9-55,3)	1	26,9 (17,7-38,7)	1	87,7 (79,6-92,8)	0,94 (0,86-1,04)
	Mulher branca (337)	45,7 (33,2-58,8)	1,43** (1,06-1,93)	21,5 (15,4-29,2)	0,73* (0,56-0,94)	14,2 (9,4-20,8)	0,85* (0,73-1,00)	93,2 (84,9-97,1)	1
	Homem negro (304)	44,5 (32,5-57,2)	1,40** (1,08-1,82)	16,5 (10,5-24,8)	0,68** (0,53-0,88)	15,7 (10,8-22,3)	0,87* (0,75-1,00)	84,0 (72,9-91,1)	0,90* (0,81-0,99)
	Mulher negra (448)	39,6 (27,4-53,2)	1,26** (1,01-1,63)	23,6 (16,0-33,3)	0,75* (0,60-0,93)	18,0 (12,7-24,8)	0,89 (0,74-1,07)	88,2 (82,2-92,4)	0,95 (0,88-1,01)
Sul (n=1.397)	Homem branco (404)	32,4 (25,3-40,5)	1	29,5 (23,3-36,5)	1	16,2 (11,2-22,7)	1	89,3 (81,7-94,0)	0,91** (0,85-0,97)
	Mulher branca (677)	34,3 (28,3-40,8)	1,03 (0,91-1,15)	23,9 (18,4-30,4)	0,93 (0,84-1,02)	18,3 (14,4-23,0)	1,03 (0,95-1,11)	97,7 (94,8-99,0)	1
	Homem negro (127)	37,9 (24,5-53,4)	1,08 (0,85-1,38)	24,9 (15,1-38,0)	0,93 (0,80-1,09)	10,0 (5,5-17,7)	0,93 (0,85-1,03)	71,1 (52,1-84,8)	0,73** (0,5-0,92)
	Mulher negra (192)	46,6 (34,8-58,8)	1,26 (0,97-1,63)	17,8 (12,3-25,0)	0,85* (0,76-0,96)	9,6 (5,7-15,7)	0,93 (0,85-1,01)	92,4 (82,5-96,9)	0,95 (0,88-1,02)

*p<0,05; **p<0,001.

As idosas do Nordeste, brancas (RP=1,31, IC 1,06-1,61) e negras (RP=1,18, IC 1,01-1,38), são mais edêntulas do que o homem branco. Já na dentição funcional (RP=0,80, IC 0,66-0,96) e na dentição completa anterior (RP=0,84, IC 0,71-0,99), a mulher negra apresentou pior condição, quando comparada ao homem branco nordestino. Foi observado, ainda, que o homem negro utiliza menos prótese (RP=0,84, IC 0,74-0,97).

No Sudeste, verificou-se desigualdade de gênero-raça na dentição funcional (RP=0,70, IC 0,57-0,85) e na dentição anterior completa (RP=0,81, IC 0,70-0,93), onde a mulher negra apresentou a pior condição. Não foram observadas desigualdades de gênero-raça em relação ao uso de prótese nesta região.

Na região Centro-Oeste, o homem branco se mantém com menor perda dentária, quando comparado com todas as categorias de gênero-raça. A mulher branca apresentou maior prevalência de edentulismo (RP=1,43, IC 1,06-1,93) e o homem negro, a menor dentição funcional (RP=0,68, IC 0,53-0,88). Sobre o uso de prótese dentária, o homem negro é menos reabilitado (RP=0,90, IC 0,81-0,99).

Por fim, a região Sul mantém o padrão de desigualdade para a mulher negra apenas na dentição funcional (RP=0,85, IC 0,76-0,96); e, de forma semelhante a outras regiões, o idoso negro utiliza menos a prótese dentária (RP=0,73, IC 0,57-0,92). Ainda em relação à reabilitação protética, o homem branco no Sul também utiliza menos prótese (RP=0,91, IC 0,85-0,97) do que a mulher branca.

## DISCUSSÃO

O presente estudo demonstrou que, embora tenham ocorrido avanços entre 2010 e 2023 para a população idosa, tais melhorias não se distribuíram de forma equitativa. O racismo e as questões de gênero atravessam o envelhecimento da população, com repercussões significativas na saúde bucal em todas as regiões do país. A idosa negra permanece como o grupo mais vulnerável em relação à perda e funcionalidade dentária, enquanto o idoso negro apresenta menor prevalência de uso de prótese.

Enquanto a redução expressiva do CPOD e do edentulismo pode indicar um avanço na política de saúde bucal e no acesso aos serviços odontológicos^1^, o aumento da dentição funcional aponta uma melhora potencial da eficiência mastigatória. Por sua vez, o aumento da dentição anterior completa reflete avanços em funções orais complementares, como a função incisal, a fonética e a estética, aspectos diretamente relacionados à autopercepção da função oral e à qualidade de vida dos idosos.

Contudo, o uso de prótese se manteve praticamente estável ao longo do período avaliado, sugerindo limitações na oferta de próteses ou persistência de barreiras de acesso à atenção especializada para pessoas idosas. Esse cenário pode estar relacionado à demanda acumulada por tratamento, decorrente da alta prevalência de perda dentária entre pessoas idosas^18^, associada à baixa oferta de outros tipos de prótese, além da prótese total^9^. O presente estudo analisou o uso de prótese em idosos que perderam 13 dentes ou mais, que demandam próteses fixas ou parciais removíveis, que por sua vez apresentam baixa oferta em todo o país^9^. Isso reflete, em parte, um histórico de modelos assistenciais centrados no indivíduo e em procedimentos de baixa complexidade^19^. Ainda, há que se considerar fortemente a influência do idadismo estrutural, cujas manifestações na saúde, em especial na saúde bucal, são evidentes^20^.

As pessoas idosas negras foram mais acometidas pela perda dentária. Esse resultado pode estar relacionado à persistência de desigualdades socioeconômicas, resultantes de processos históricos de exploração desta população^11^ ^,^ ^21^, com a consequente menor disponibilidade de recursos econômicos e estruturais e dificuldade de acesso aos serviços odontológicos^11^. Destaca-se também o caráter subjetivo da autoavaliação em saúde bucal, que exerce impacto negativo na percepção das necessidades e dos problemas bucais, refletindo um conjunto de experiências históricas, sociais e culturais pela população negra^11^ ^,^ ^22^ ^,^ ^23^.

Outro aspecto relevante refere-se à forma como a atenção odontológica tem sido ofertada à população negra. Evidências apontam que esses indivíduos apresentam maior probabilidade de serem submetidos a procedimentos de urgência ou extrações dentárias, em detrimento de abordagens conservadoras e restauradoras^24^. Essa diferença pode estar relacionada à influência da raça/cor da pele na decisão clínica, revelando o racismo nas condutas profissionais^22^. O racismo estrutural se manifesta também nas práticas e decisões em saúde^22^ ^,^ ^24^, contribuindo para a manutenção das iniquidades no cuidado odontológico^25^.

A análise interseccional de raça/cor-gênero na saúde bucal evidenciou que a idosa negra apresenta os piores indicadores de perda dentária, enquanto o negro é o menos reabilitado. Essas diferenças refletem o peso histórico das desigualdades de raça e gênero, associadas à renda, dupla jornada e violência à mulher negra^26^. Esses fatores operam como determinantes sociais estruturais da saúde, influenciando a exposição cumulativa ao adoecimento bucal ao longo do curso de vida e o acesso oportuno e resolutivo aos serviços odontológicos.

Apesar de apresentarem maior perda dentária, as mulheres tendem a buscar mais serviços odontológicos e usarem mais prótese dentária do que os homens^23^ ^,^ ^27^, possivelmente por maior autopercepção e atenção à saúde^23^ ^,^ ^28^. Contudo, isso não se traduz em melhores condições bucais^29^, como evidenciado no presente estudo, em consonância com a literatura^28^ ^,^ ^30^ ^,^ ^31^. Esse cenário é exacerbado quando ocorre a sobreposição dos marcadores de raça/cor e gênero, resultando em maior vulnerabilidade para idosas negras, de forma semelhante a outro estudo^6^ que também identificou maior perda dentária para mulheres negras.

Já entre os homens, a menor procura por serviços pode estar relacionada a barreiras de horário, medo do diagnóstico e padrões culturais de masculinidade que desestimulam o autocuidado^23^. Essas diferenças de gênero evidenciam os limites estruturais do cuidado tanto no adoecimento quanto nas oportunidades de tratamento e reabilitação. Na perspectiva interseccional, o homem negro percebe a necessidade de saúde com base em vivências sócio-históricas-culturais, colocando-o em situação de desvantagem em relação ao homem branco no acesso à reabilitação protética. Somado a isso, a raça tem influência na escolha de tratamento pelo dentista^32^. Por exemplo, outro estudo^29^ identificou que a pessoa negra com dente cariado tinha uma chance 50% menor de ser encaminhada para tratamento protético.

As desigualdades regionais identificadas demonstram que o local de residência exerce forte influência sobre as condições de saúde bucal na velhice. As regiões Sudeste e Sul, assim como residir nas capitais, revelaram as melhores condições de saúde bucal^7^ ^,^ ^8^ ^,^ ^19^. Essa diferença pode ser explicada, em parte, pela distribuição desigual de profissionais de odontologia no país, uma vez que, embora o número total de dentistas seja elevado, sua concentração é maior nas regiões mais desenvolvidas e no setor privado^33^. Essa disparidade cria barreiras significativas ao acesso aos cuidados preventivos e reabilitadores, contribuindo para a manutenção de iniquidades territoriais e maior vulnerabilidade das pessoas idosas que vivem fora dos grandes centros.

Além disso, fatores socioeconômicos e demográficos distintos entre as regiões influenciam o processo de envelhecimento e o uso dos serviços odontológicos, reforçando o caráter desigual do cuidado^34^ ^,^ ^35^ ^,^ ^36^. Na perspectiva da oferta de prótese dentária, observa-se um crescimento desigual do número de Laboratórios Regionais de Prótese Dentária (LRPD)^37^ e variação na produção de próteses entre as regiões do Brasil^21^, revelando disparidades regionais da política de saúde bucal.

Assim, mesmo com a ampliação das redes de atenção no país, persistem lacunas no acesso a serviços odontológicos para determinados grupos populacionais^36^, especialmente a população negra. Um estudo identificou a presença de desigualdade racial na entrega de prótese total no país, visto que, mesmo em municípios com maior proporção da população negra, maior oferta de equipes de saúde bucal e Centro de Especialidade Odontológica (CEO), observou-se que a entrega de prótese dentária foi maior para a população branca^38^.

Na perspectiva interseccional por região, o padrão nacional de maior perda dentária para a mulher negra e menor uso de prótese para o homem negro se mantém, entretanto, peculiaridades regionais são evidenciadas na análise interseccional territorial.

Na Região Norte, apesar dos piores indicadores médios de saúde bucal, não foram observadas desigualdades significativas segundo gênero-raça. Esse achado pode refletir um contexto de elevada vulnerabilidade estrutural, no qual barreiras geográficas, menor oferta de serviços odontológicos e desigualdades territoriais históricas impactam de forma generalizada a população idosa, atenuando a diferenciação dos desfechos entre os grupos analisados. Enquanto a região Sudeste não apresentou desigualdades de gênero-raça na prevalência de edentulismo e no uso de prótese, na região Sul não se observou desigualdade no edentulismo. Em contraste, na região Centro-Oeste, o homem branco se destaca em privilégio quanto a menor perda dentária e melhor dentição funcional em relação a todas as outras categorias de gênero-raça, revelando uma desigualdade mais expressiva que nas outras regiões. Esse cenário reflete a permanência de desigualdades estruturais históricas e destaca a necessidade de políticas e estratégias regionalizadas que considerem as especificidades sociais, econômicas e culturais de cada território.

Analisar essas demandas sob perspectivas individuais, contextuais e raciais é essencial para o planejamento de políticas públicas mais justas e voltadas à equidade em saúde bucal, garantindo prioridade aos grupos historicamente vulnerabilizados^11^ ^,^ ^12^ ^,^ ^19^. Contudo, considerando o histórico de elevada perda dentária e baixa reabilitação entre as pessoas idosas brasileiras, é provável que melhorias significativas nesse quadro ocorram apenas com as novas gerações, que tendem a envelhecer com melhores condições bucais.

O presente estudo destaca a importância de incorporar a perspectiva interseccional nas políticas de saúde bucal dirigidas à população idosa, e que esta dialogue com a Política de Saúde Integral da População Negra^39^, no sentido de combater o racismo sistêmico no campo da saúde bucal e todas as formas de discriminação em qualquer espaço do viver^40^. É necessário que políticas públicas com ações afirmativas e programas educativos voltados para o benefício de mulheres negras sejam mantidos e repensados, a fim de assegurar acesso universal e equitativo, em consonância com os princípios do SUS^6^ ^,^ ^36^. A consideração de marcadores de gênero, raça e território no planejamento, monitoramento e na avaliação das intervenções pode contribuir para reduzir desigualdades e fortalecer o SUS.

Entre as limitações deste estudo, tem-se a não inclusão da população de 60 a 64 anos no SB Brasil, excluindo parte da população idosa negra, visto que sua expectativa de vida é menor que a da população branca. Outra limitação foi a análise da categoria negro, o que pode ocultar heterogeneidades internas entre pretos e pardos, assim como a análise da dentição funcional sem considerar os pares oclusais e sem discriminar o uso dos diferentes tipos de prótese dentária.

Apesar dos avanços na saúde bucal dos idosos brasileiros, persistem desigualdades marcadas pela interseção entre gênero, raça e território, com impacto mais severo sobre mulheres negras e indivíduos residentes em regiões historicamente desfavorecidas. A manutenção dessas iniquidades revela os limites das políticas universais e reforça a necessidade de estratégias afirmativas no âmbito do SUS, que considerem especificidades socioculturais e territoriais dos diferentes grupos populacionais.
